# Risk-Based Monitoring in Clinical Trials: Increased Adoption Throughout 2020

**DOI:** 10.1007/s43441-022-00387-z

**Published:** 2022-03-02

**Authors:** Nicole Stansbury, Brian Barnes, Amy Adams, Ruth Berlien, Danilo Branco, Debby Brown, Paula Butler, Lauren Garson, Debra Jendrasek, Garrett Manasco, Nycole Ramirez, Nelly Sanjuan, Gillian Worman, Anina Adelfio

**Affiliations:** The Association of Clinical Research Organizations (ACRO), Washington, DC USA

## Abstract

With the emergence of new technologies for data collection, the continued impact of the COVID-19 pandemic, and the increasing number of partially or fully decentralized clinical trials (DCTs), the importance of risk-based monitoring (RBM) and the larger risk-based quality management (RBQM) framework in clinical trial management is increasing. RBM and RBQM focus on the detection of events or trends that impact trial quality in terms of participant safety and data integrity. In 2019, the Association of Clinical Research Organizations (ACRO) began a landscape survey of RBM/RBQM implementation in ongoing clinical trials. Initial results of this survey, representing full-year data for 2019, were reported previously. Here, we present full-year landscape data for 2020 drawn from 5,987 clinical trials ongoing at the end of 2020, including 908 new studies started that year. Of these trials, 77% implemented at least one RBM/RBQM component, an increase from 47% for studies ongoing at the end of 2019. We also observed increased implementation for three of the five RBM components included in the survey. Centralized monitoring decreased nominally in 2020 compared with 2019. Although the percentages of 2020 trials incorporating reduced source data verification (SDV) and reduced source data review (SDR) increased from 2019 to 2020, these numbers are still low considering the large percentage of trials implementing at least one RBQM component. In the current clinical trial landscape, as more DCTs are launched and new data collection technologies are implemented, there remains a pressing need for greater use of centralized monitoring coupled with reductions in SDR/SDV and, ultimately, greater adoption of RBM and RBQM.

## Introduction

Risk-based monitoring (RBM) in clinical trials focuses on detecting, addressing, preventing, and mitigating risks that could compromise critical trial processes, patient safety, or data integrity. This more efficient approach to trial monitoring is an integral part of the risk-based quality management (RBQM) framework, described by the European Medicines Agency (EMA) in 2013 as “a systematic process put in place to identify, assess, control, communicate and review the risks associated with the clinical trial during its lifecycle.” [1]. Despite a growing body of evidence showing the benefits of RBM in clinical trial management, adoption has been slow and implementation incomplete [2, 3]. The COVID-19 pandemic, however, has highlighted the benefits of implementing RBM alone and as part of a broader RBQM approach.

The Association of Clinical Research Organizations (ACRO) is a trade association of global contract research organizations (CROs) and technology companies. ACRO’s mission is to advocate to regulators and policymakers on behalf of the clinical research industry, educate stakeholders, and help inform policy. We previously reported results from ACRO’s landscape survey on the implementation of RBM/RBQM components across 6513 clinical trials ongoing at the end of 2019, as well as additional data on trial execution during the first half of 2020, at the onset of the COVID-19 global pandemic [3].

Among the most significant findings of the landscape survey was that prior to the pandemic, only 22% of trials included at least one RBM component, with each individual component being implemented in just 8–19% of trials [3]. There was, however, a rapid shift from 82% trials using on-site monitoring in February 2020 to 93% of trials using remote monitoring in April 2020, corresponding with the first wave of the pandemic. At that time, ACRO members rapidly gathered data on protocol deviations, using this information as a proxy measure of monitoring effectiveness and trial quality. One CRO reported little change in the number of protocol deviations detected each month from February through June 2020. This indicated that the rapid transition to off-site/remote monitoring did not compromise the ability to identify protocol deviations, despite the reduction in on-site monitoring activities.

One unanswered question from the earlier survey was whether the shift in monitoring practices during the early months of the pandemic would be maintained long term. ACRO conducted a follow-up survey using the same methodology to collect new data on RBM implementation through the end of 2020. Here, we present these data and discuss new insights gained into the evolution of clinical trial monitoring during an unprecedented and unexpectedly prolonged pandemic disruption that has impacted almost every aspect of daily life. We also explore the implications of our findings for clinical trial management post-pandemic based on our experience with RBM and RBQM implementation.

## Methods

For the previously published 2019 landscape analysis, seven ACRO member companies responded to a survey of RBQM practices in clinical trials where project management and/or clinical monitoring were within scope of the companies’ services. Two of the companies subsequently merged, so the 2020 survey update included data from six companies. It should be noted that one of the companies participating in the merger did not provide data for the update.

As previously reported, an independent outside vendor collected, blinded, aggregated, and analyzed the data [3]. The original dataset captured trials that were ongoing as of December 31, 2019, including studies initiated in 2019 and multi-year studies initiated in years prior. The updated dataset captured trials that were ongoing as of December 31, 2020, including 908 studies initiated in 2020 and multi-year studies initiated in years prior.

To better understand the RBM landscape, companies participating in the survey were asked to provide data showing how many of their trials implemented one or more of the eight RBQM components: initial cross-functional risk assessment, ongoing cross-functional risk assessment, quality tolerance limits (QTLs), key risk indicators (KRIs), centralized monitoring, off-site/remote-site monitoring, reduced source data verification (SDV), and reduced source data review (SDR). While all eight components are part of the RBQM framework, the last five make up the critical components of RBM. Component definitions were formulated by the authors to provide relevant benchmarks to support data collection, ensuring that data submissions were consistent in the survey responses [3].

With increasing adoption of RBM and RBQM and the emergence of technologies supporting remote monitoring, data collection, data processing, and data analysis, more trials are using decentralized clinical trial (DCT) approaches where trial activities take place away from traditional trial sites. For this reason, participating companies were also asked to provide information on implementation of DCT components in the 2020 survey; however, due to variation in data collection practices and the lack of standardized metrics to track adoption, the survey did not capture sufficient DCT component data to support meaningful analysis.

## Results

**Fig. 1:**
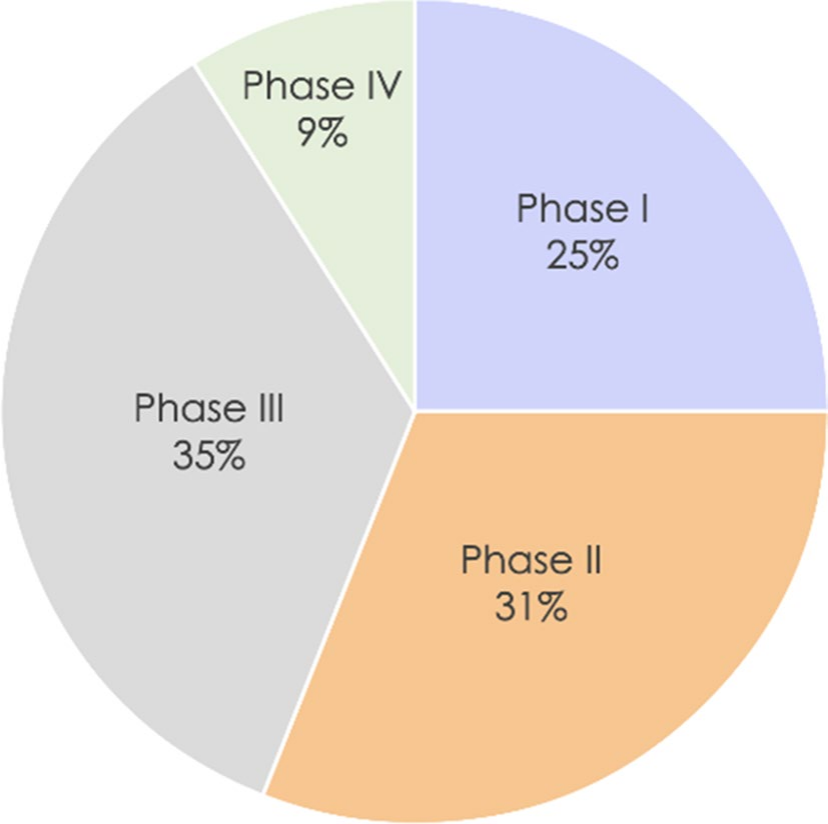
*Clinical Trials in the Landscape Survey by Trial Phase*. Shown here are the percentages of clinical trials in the 2020 data set for trial phases I–IV

The survey respondents included six different CROs providing data on 5,987 studies that were ongoing in 2020. Of these, 908 were new studies started in 2020. Our sample included a diverse set of studies representing all four clinical trial phases, with phase I accounting for 25% of the included studies, phase II accounting for 31%, phase III accounting for 35%, and phase IV accounting for 9% (Fig. 1).

**Fig. 2:**
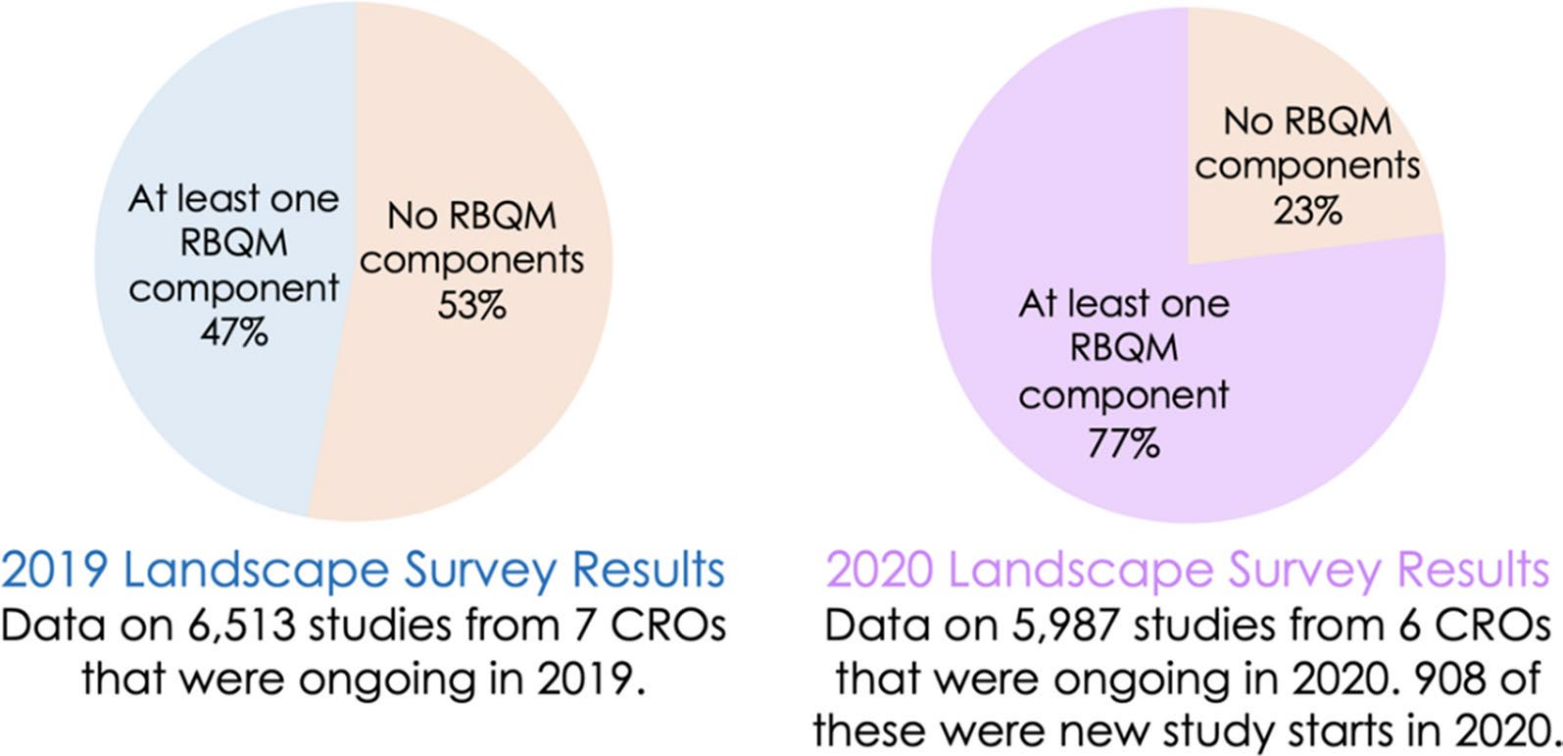
*RBQM Implementation in 2019 and 2020*. Graph shows the percentage of ongoing studies that implemented at least one of the eight RBQM components and the percentage of studies that did not implement any of the RBQM components

Of the nearly 6000 studies included in the 2020 data set, 77% had at least one RBQM component, whereas 23% did not. For comparison, the previous year’s data revealed that 47% of studies had at least one RBQM component, and 53% implemented none (Fig. 2). This represents a 30-percentage point increase year-over-year.

**Fig. 3:**
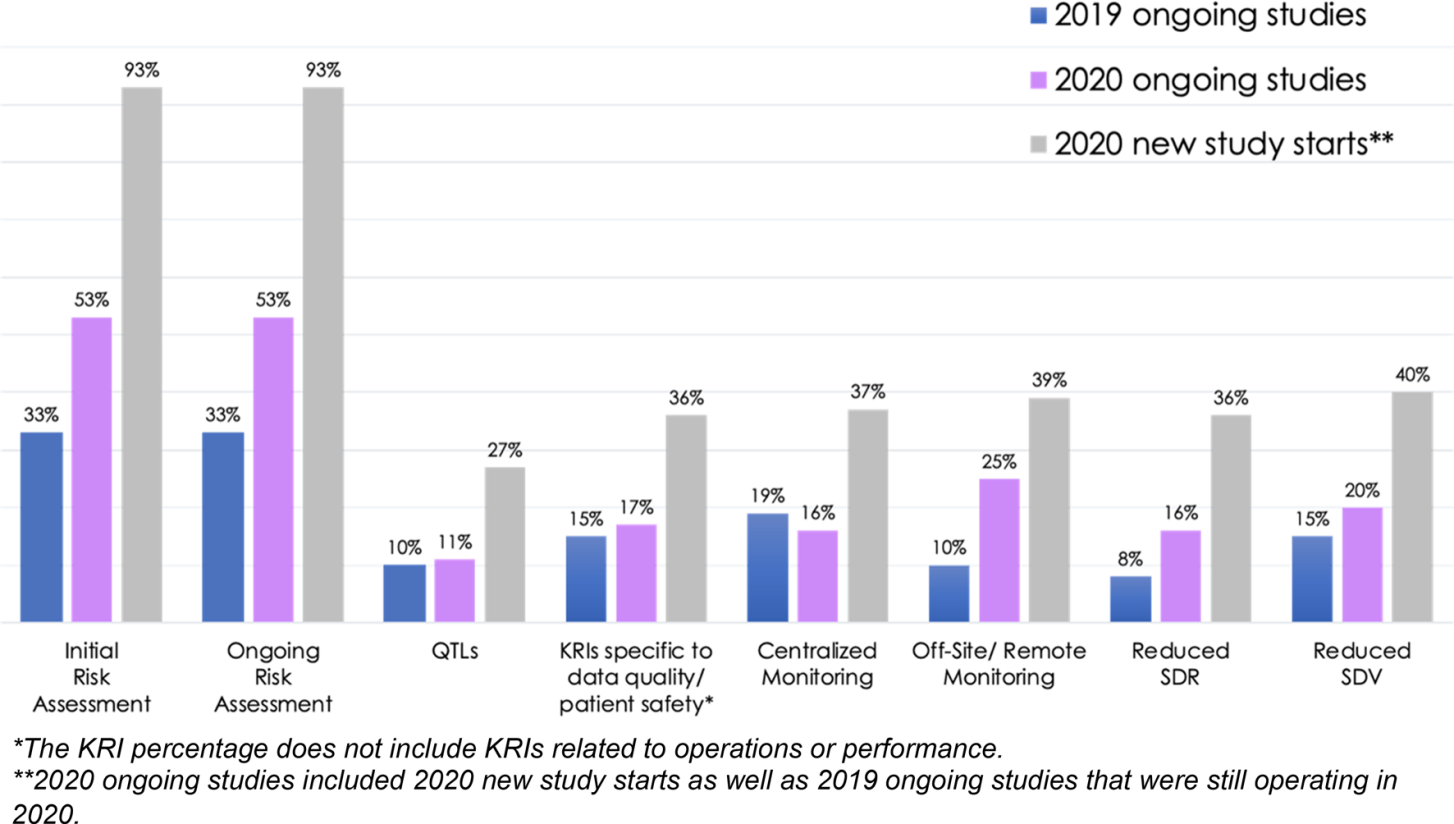
*2019–2020 Landscape of RBM/RBQM Component Implementation in Clinical Trials*. Data represent the percentage of all trials included in the 2019 and 2020 surveys that implemented each component and not just the subset of studies that have at least one RBM component

Implementation of three RBM components—off-site/remote monitoring, reduced SDR, and reduced SDV—increased from 2019 to 2020 (Fig. 3, blue and purple bars, respectively). There was little year-over-year change in KRIs and centralized monitoring. Most notably, implementation of two RBQM-specific components—initial and ongoing risk assessment—which contributes to a more holistic approach to clinical trial management, each increased by 20 percentage points from 2019 to 2020.

The components implementation data for studies started in 2020 (Fig. 3, gray bars) show clear trends of increased RBM/RBQM implementation. All components were implemented in a higher percentage of new studies started in 2020 compared with either all 2020 ongoing studies or all 2019 ongoing studies (note that 2020 ongoing studies included 2020 new study starts as well as 2019 ongoing studies that were still operating in 2020). The five RBM components were implemented in similar percentages of studies started in 2020 (36–40%), though it should be noted that studies implementing one RBM component did not necessarily implement any of the others. For RBQM components, there was a large increase for 2020 new study starts in risk assessment implementation compared with all 2020 ongoing studies or 2019 ongoing studies. QTL implementation was also more than 2.5 × higher in 2020 new study starts than in all 2020 ongoing studies or 2019 ongoing studies.

## Discussion

The landscape survey data show year-over-year growth in RBM/RBQM component implementation, but adoption rates among all ongoing studies for the individual components and the RBM/RBQM frameworks are still low (Figs. 2 and 3). The 2020 new study start data show sustained increases in RBM adoption. Our data demonstrates that sponsors, CROs and technology vendors are increasing RBM and RBQM adoption, and this was potentially accelerated during the COVID-19 pandemic, as initially observed in the previous survey [3]. Based on the data and our own experiences, we identified three components that are critical to future uptake of RBM/RBQM: risk assessment, centralized monitoring, and QTLs.

### Risk Assessment

The dramatic 20% increase in risk assessments from 2019 to 2020 is in part driven by risk assessment implementation in 2020 new study starts [4–6]. Regulatory support of RBM/RBQM is not new, but the disruptive effects of the pandemic accelerated adoption in a way that previous regulatory and industry advocacy did not [1, 6]. The increasing implementation of DCTs—which often generate large quantities of data through multiple data sources that are best managed through centralized monitoring—likely also played a role in the increased risk assessment activities. RBM/RBQM approaches are particularly suitable for managing DCTs.

One set of guidelines that has played a pivotal role in RBM/RBQM adoption come from the International Council for Harmonisation of Technical Requirements for Pharmaceuticals for Human Use (ICH). The ICH Guideline for Good Clinical Practice E6(R2) released in 2016 contained recommendations on RBM that are best addressed within the larger RBQM framework [7]. In a draft update of the ICH E6 Principles that was released in March 2021 as part of the updates being made to ICH E6(R2) in advance of releasing the full ICH E6(R3) guideline, one of the most significant messages is alignment with the ICH E8(R1) General Considerations for Clinical Studies guideline (draft released in 2019, with guideline fully adopted in October 2021) regarding use of quality-by-design principles in clinical trial planning [8, 9]. The ICH E6(R3) draft also suggests innovative technologies may be used to improve trial quality and stresses the consideration of how potential risks to participant safety or data integrity impact critical-to-quality factors of the trial. The full ICH E6(R3) guideline will make it clear that the overall quality of a trial is driven proactively by designing quality into the study protocol and processes, with appropriate and fit-for-purpose use of technology. These principles should be applied during the early planning stages and across trial operations.

Risk assessment is the foundation for greater adoption of RBM/RBQM and the other components that make up these frameworks. Defining critical data and processes and their associated risks early during trial planning promotes the uptake of other components, particularly centralized monitoring and QTLs.

### Centralized Monitoring Improves Data Insights

We expected to see an increase in centralized monitoring in 2020, as trials had to adapt to the realities of the pandemic. Instead, we saw a 3-percentage-point decrease in centralized monitoring implementation for all trials, although 2020 new trial starts were two times more likely to implement centralized monitoring compared with all 2020 ongoing studies (Fig. 3). Centralized monitoring enables the reduction of SDV/SDR by allowing organizations to focus on the datapoints that matter. This more holistic view of the clinical trial data allows SDR/SDV efforts to be targeted based on the output of the centralized monitoring process, increasing monitoring efficiency. Implementing centralized monitoring without reduced SDR/SDV results in utilization of resources that could be better deployed elsewhere.

Centralized monitoring allows data visualization that provides insights beyond those that can be gained on-site from the perspective of the investigator or the clinical research associate (CRA) through SDR/SDV. Unfortunately, many organizations continue to conduct very costly 100% SDV/SDR on-site, even though the practice typically uncovers few errors that meaningfully impact data quality or patient safety [10]. This is often done in the belief that if all errors are not captured, critical-to-quality errors will be missed, even though targeted RBM approaches may be more likely to detect critical errors while not triggering unnecessary review of those errors that don’t affect trial quality. A major advantage of centralized monitoring is that it allows real-time evaluation of data, making early interventions possible to ensure participant safety and data integrity.

**Fig. 4:**
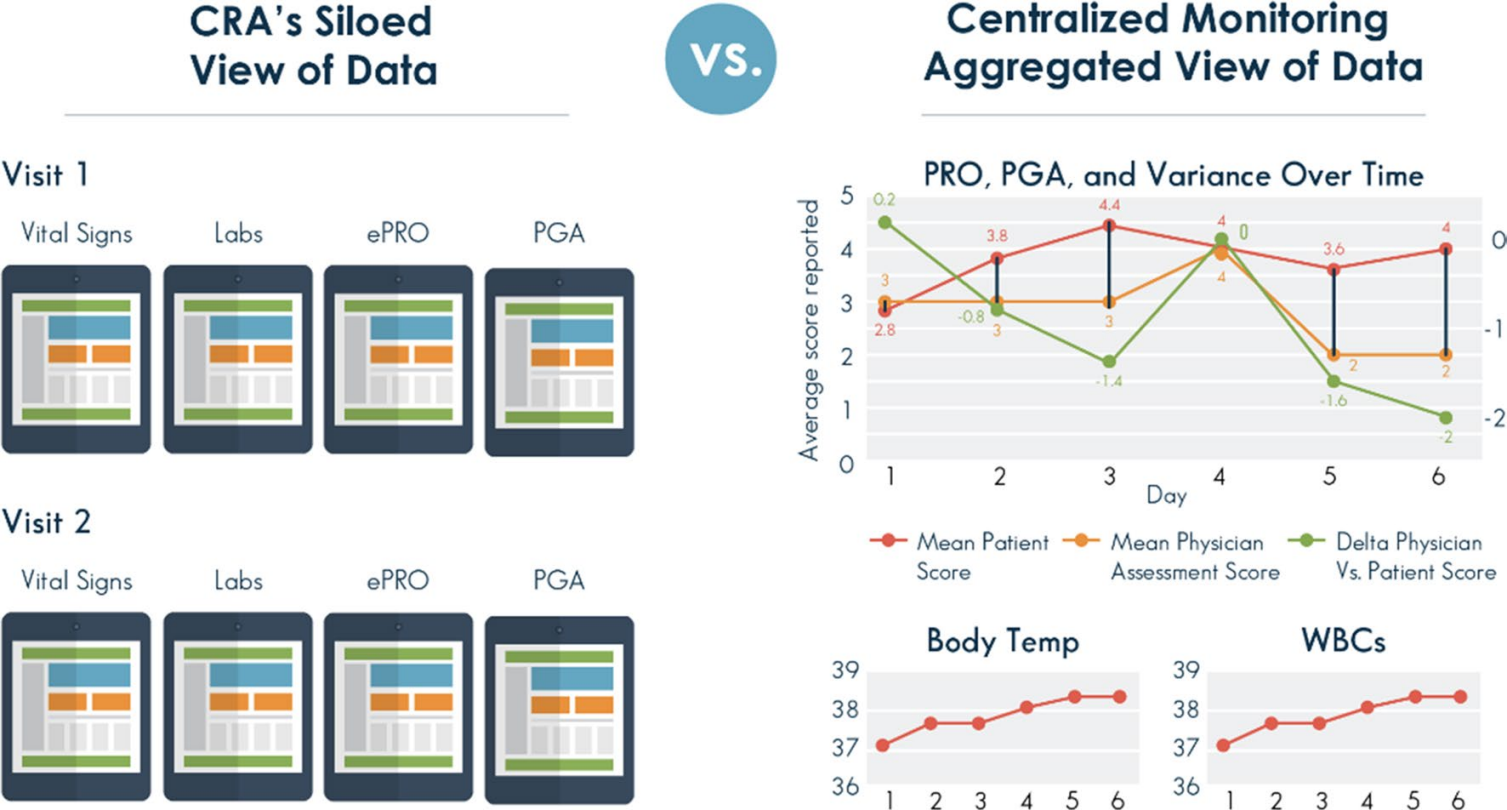
*Monitoring of Siloed Data Versus Centralized Monitoring with Visualization of Aggregated Data*. Centralized monitoring allows clearer identification of trends or aberrations in clinical trial data compared with monitoring by a CRA, who has a more siloed view of the data

With the increasing number of fully or partially decentralized trials and the emergence of new technologies leading to use of diverse data sources and collection methods in a single trial,100% SDR/SDV becomes ineffective to meet monitoring needs because it does not capture associations between multiple data sources. For example, a trial investigator may oversee the medical care for subjects but not have access to data coming in from electronic patient surveys or a wearable device monitoring heart rate. Digital data gathered remotely or directly from the source is usually not included in the patient’s on-site or electronic medical record and is, therefore, usually not subject to SDV. Centralized monitoring pulls together data collected from different sources to produce a holistic view by aggregating this information to identify compliance deviations and trends critical to subject safety and data integrity [Fig. 4]. This type of analysis can be done at the patient level, site level, or study level and can reduce the need for on-site monitoring.

The principles of trial monitoring set out in ICH E6(R2) are: (1) to protect study participants’ rights, well-being, and safety; (2) ensure data integrity; and (3) ascertain protocol and regulatory compliance [7]. Centralized monitoring, used in 16% of 2020 studies, is most efficient when combined with reductions in SDR and SDV, which only occurred in 16% and 20% of studies, respectively. Failure to reduce SDV/SDR when implementing centralized monitoring results in duplication of effort. Because centralized monitoring reduces the need for on-site review and source verification activities, continued reliance on 100% SDR/SDV undermines its value and, ultimately, the use case. Without greater acceptance of reduced SDR/SDV, implementation of centralized monitoring and adoption of RBM/RBQM will continue to be lower than is optimal.

Based on the 2019 and 2020 landscape surveys, as well as our own experience in clinical trial management, reducing SDV and SDR is crucial for implementation of risk-based approaches to monitoring and trial management. This is why reduced SDV and reduced SDR are two of the five components that define RBM and two of the eight components that define RBQM. Beyond these two frameworks, however, an overall trend toward greater use of technology, which drives decentralized data collection in clinical trials, demonstrates the need to reduce SDV/SDR through remote monitoring as part of a centralized monitoring strategy. Increased use of centralized monitoring and decreased reliance on SDR/SDV activities is more compatible with the propagation of data by many different data sources, as is seen more frequently in newly started clinical trials, particularly DCTs.

### Quality Tolerance Limits: When to Evaluate, When to Act

QTLs are early and important signals of data integrity or safety concerns that may jeopardize the overall study success. When these predetermined thresholds for trial parameters are reached, that triggers evaluation to determine if intervention or mediation is necessary to preserve the quality of the trial. Our data show implementation of QTLs in clinical trials is still very low, with just 11% of trials in the 2020 landscape survey having QTLs. This may be because their use is still evolving. QTLs are, however, vital to making a holistic RBQM approach effective because they affect ongoing decisions about trial management. Improving QTL adoption should be an industry goal. To do so, it would help to have more clarity on implications within a clinical study report (CSR) if there are excursions noted. Sponsors may be wary of implementing QTL thresholds where excursions are required to be noted in the trial’s CSR out of fear this could impact the approval of their product. The fact that 27% of 2020 new study starts included QTLs indicates some progress is being made, though it remains to be seen what the long-term trajectory of QTL adoption will look like.

### Putting it Together: Remote Data Collection, Centralized Monitoring, and Decentralized Clinical Trials

The rising complexity of clinical trial protocols, the increase in the types and volume of patient-reported data, and the challenges of the COVID-19 pandemic have heightened attention and interest in RBQM and DCTs. The defining feature of DCTs is that “some or all trial activities take place outside of traditional trial sites.” [11]. Although it may seem contradictory to pair the concepts of trial decentralization and centralized monitoring, the relationship is actually synergistic. For a trial to be less location-dependent and less reliant on on-site activities, there must be centralization of data collection and analysis. An understanding of all sources for data capture in a clinical trial and the centralization process is key to conducting DCTs. Although there are relatively few fully decentralized trials, in our experience, the majority of currently ongoing trials have at least one DCT component.

Decentralized data collection is achieved through a variety of mechanisms, including mobile technologies, sensors, mobile healthcare providers, electronic data capture (EDC), and third-party vendors such as central labs. It is critical to proactively outline during protocol development how data will flow during the clinical trial and identify potential critical-to-quality risks. As part of the initial risk assessment, it is imperative to identify potential risks associated with this data flow and also how to use data to monitor risks specific to other trial activities. Traditionally, sites entered clinical trial data through EDC, as SDV is performed. SDV is not required for data collected electronically directly from participants, but targeted SDR is still valuable. In trials with both on-site data collection recorded directly by a doctor or nurse and electronic data capture flowing to centralized monitoring, SDV should be targeted only at the data where errors might be introduced during collection (such as transcription errors or incomplete data entry from medical charts). This underscores the pivotal role of centralized and remote monitoring to ensure the quality and integrity of the data while also reducing SDV and SDR.

In our 2020 survey, we asked respondents to report implementation of the following DCT components:

- eConsent/eSignature
- Direct to/from patient shipments
- Home health visits
- Telemedicine
- eCOA (electronic clinical outcome assessment)/ePRO (electronic patient reported outcome)
- Connected devices/ Digital endpoints
- Remote review of source documents

The tracking and reporting of DCT components are maturing and were not robust enough to make any concrete conclusions from the current data analysis. Future surveys will attempt to better track their implementation, and we encourage the industry at large to track and report use of DCT components going forward.

As technology for collecting data outside of a traditional trial site (e.g., gathered directly from patients using a wearable device or entered into a mobile device by a nurse during a home visit) becomes more accepted, adoption of decentralized methods will increase as more investigators/study managers recognize their value. The impact of technology on clinical trial operations has been tremendous; however, when technology is applied to clinical trial conduct, it must be adapted to fit individual participant circumstances and the particular trial design [8]. This highlights the core interaction between the application of technology with quality-by-design principles, which can be seen in the interaction between DCT implementation and RBM/RBQM adoption. Risk assessments and centralized monitoring need to become standard practice as more new trials implementing DCT components are launched. It is important that adoption of DCT methods does not outpace adoption of centralized monitoring methods because older approaches to monitoring are generally not compatible with DCTs.

Technology is driving decentralization of data collection and monitoring, even in trials that would not be considered DCTs. But decentralized components cannot be successfully implemented without centralized monitoring due to the velocity and volume of data generated—as well as the myriad of different data sources now in use. For this reason, data collection and monitoring must be centralized to see the full picture, which is made possible by aggregating the inputs from different data streams. This new reality also renders site-based methods such as 100% SDV/SDR insufficient, inappropriate and ineffective.

## Conclusion

The introduction of technology into the ICH principles of Good Clinical Practice highlights the integral role that technology will play in the future of clinical trials [8]. We have already seen these principles put into action through trial adaptations during the pandemic. ICH E6(R3) will outline future expectations regarding technology and systems, based on the acceptance by regulatory and industry leaders of new data collection and analysis techniques to support robust, rapid, and complex clinical trials. Without these technologies, patient participation in clinical trials and oversight activities outside the clinical investigative site would not have been possible during the pandemic. That these changes in trial activities were so effective reflects the modernization of clinical trial operations that was already taking place for years prior to the pandemic.

In our previous analysis of landscape survey data, we stated the goal of future research would be to determine if the increase in RBM/RBQM implementation would be sustained. Now that we are further into but still not at the end of the pandemic, our new data show that the shift to more risk-based approaches is being maintained and, based on the data from 2020 new study starts, appears to be accelerating. There is still a need, however, for better adoption of RBM/RBQM, particularly the greater use of centralized monitoring with reductions in SDR/SDV as more and more clinical trials using DCT components are launched.

In future updates of the landscape survey, we plan to gather more data on RBM and RBQM implementation—and how the industry is changing to embrace these strategies as they develop over time—as well as the use of DCT components, which may be a vital driver of RBM adoption going forward.
